# The Effect of Selected Phosphate-Solubilizing Bacteria on the Growth of Cotton Plants in Salinized Farmlands

**DOI:** 10.3390/microorganisms13051075

**Published:** 2025-05-05

**Authors:** Tong Wang, Yan Sun, Hong Huang, Ziwei Li, Hua Fan, Xudong Pan, Yiwen Wang, Yuxin Cao, Kaiyong Wang, Le Yang

**Affiliations:** 1 Agricultural College, Shihezi University, Shihezi 832000, China; t_sing@163.com (T.W.); sunyan72@xjshzu.cn.com (Y.S.); lzw_17301204609@163.com (Z.L.); fanhua@shzu.edu.cn (H.F.); pxd0801@163.com (X.P.); 15700974352@163.com (Y.W.); cyx18711789295@163.com (Y.C.); 2 State Key Laboratory of Efficient Utilization for Low Grade Phosphate Rock and Its Associated Resources, Guiyang 550000, China; hong.huang@live.com

**Keywords:** phosphate-solubilizing bacteria, soil fertility, soil microorganisms

## Abstract

The utilization rate of phosphorus fertilizer is low in Xinjiang, China, due to the fact that phosphorus is easily fixed by the widely distributed lime soil, leading to the limited contribution of phosphorus fertilizer to crop yield and a decline in crop quality. Phosphate-soluble bacteria can convert insoluble phosphates in the soil into soluble phosphates, playing an important role in soil phosphorus circulation and plant growth. In this study, two bacteria with strong phosphate-solubilizing ability, *Enterobacter hormaechei* (P1) and *Bacillus atrophaeus* (P2), were selected from severely salinized soils in Xinjiang, China. The taxonomic status of the strains was determined by analyzing the colony morphology and 16S rRNA gene sequence similarity. Then, the content of organic acids and the activity of acid phosphatase and phytase in the P1 and P2 fermentation broths were measured. Finally, field experiments were conducted in 20 April–2 October 2023 in Wulanwusu, Xinjiang, China, to analyze the effects of phosphate-solubilizing bacterial agents (P1, P2, and P3 (P1 + P2)) on soil physicochemical properties, microbial diversity, and cotton yield. The results showed that both P1 and P2 could significantly solubilize phosphates and produce indole-3-acetic acid (IAA), lactic acid, and tartaric acid. In the cotton field under phosphorus fertilization, the cotton yield of P1, P2, and P3 treatments increased by 10.77%, 8.48%, and 14.00%, respectively, compared with no bacterial agent treatment (CK) (*p* < 0.05). In addition, the application of phosphate-solubilizing bacterial agents also significantly increased the content of available nutrients and the abundances of *Acidobacteria*, *Bacteroidetes*, *Fusarium*, *Bacteroidetes*, and *Verrucobacteria* in the soil compared with CK. In summary, inoculating with phosphate-solubilizing bacteria could promote cotton growth and yield formation by increasing soil available nutrients and altering soil microbial communities. This study will provide a basis for the efficient utilization of phosphorus resources and sustainable agricultural development.

## 1. Introduction

The global salinized land area exceeds 830 million hectares, accounting for 67.3% of the earth’s land area, and is increasing at a rate of 1.0 × 10^6^–1.5 × 10^6^ hectares per year [1]. In addition, the productivity of about 5.70 billion hectares of farmland worldwide is limited by insufficient available phosphorus in the soil [2]. Phosphate ions are prone to form insoluble calcium phosphate, magnesium phosphate, and other compounds with high concentrations of calcium, magnesium, and other cations in salinized soils, resulting in phosphorus being fixed in the soil and difficult to be absorbed and used by plants.

In 2023, Xinjiang’s cotton planting area was 2.49 million hectares, with a total output of 5.11 million tons, accounting for more than 70% of China’s total cotton output [3,4]. Phosphorus is one of the indispensable macro elements in plant growth. It can significantly increase cotton yield and quality by promoting cotton root growth, photosynthesis, boll development, and enhancing stress resistance. However, there are large areas of salinized soil in Xinjiang, China. Salinized arable land accounts for 42.07% of the total arable land in Xinjiang. In addition, the widely distributed lime soil (calcium carbonate > 15%) in Xinjiang causes most of the phosphorus to be quickly adsorbed and fixed into unavailable forms after phosphorus fertilization, resulting in 95% of the phosphorus being unable to be directly absorbed by crops [5]. To solve this problem, Xinjiang farmers always apply a large amount of phosphorus fertilizer. Excessive phosphorus application not only increases production costs but also causes a waste of phosphorus (only 15–20% of phosphorus is used by plants) and environmental pollution [6,7,8]. It should be noted that phosphate is a non-renewable resource, and half of the existing global phosphate reserves will be depleted within 50–100 years [9,10]. Therefore, how to increase the utilization rate of chemical phosphorus fertilizer and reduce the application rate while maintaining cotton yield is an urgent issue to be solved, which is the key to reducing environmental pollution and achieving sustainable agricultural development in Xinjiang.

Phosphate-solubilizing microorganisms (PSMs) refer to functional microorganisms that can convert and decompose insoluble phosphates into soluble and bio-available phosphates. The PSMs include phosphate-solubilizing bacteria (PSBs), fungi, and actinomycetes. Among them, PSBs account for 1–50% of the total PSMs and about 40% of the cultivable bacteria in soil. Therefore, PSBs are widely used currently [11,12]. Most PSBs can produce indole-3-acetic acid (IAA), promoting plant cell growth. In addition, the released microbial metabolites and organic acids during PSBs metabolism are crucial for the solubilization of phosphorus in soil [13,14]. The application of PSBs is currently an important measure to increase the available phosphorus content in farmland soil. PSBs can be mixed with mineral phosphorus and organic phosphorus to increase phosphorus fertilizer use efficiency in farmlands [15], which provides a new approach for increasing soil phosphorus availability.

In addition, Muhammad et al. [16] found that cotton plant height, average boll weight, net photosynthetic rate, fiber length, and seed cotton yield increased after inoculating with rhizosphere bacteria and PSBs. Haider et al. [17] also found that the leaf area index, photosynthetic rate, and seed cotton yield significantly increased after inoculation with the PSBs selected from the cotton rhizosphere. However, most of the previous research on the impact of PSBs on plants is concentrated in maize and wheat, and the impact on cotton is less studied. In addition, most of the studies on PSBs are concentrated in neutral soils, while few studies focus on the PSBs isolated from saline soils. In-depth study of the mechanism of PSBs impacting cotton growth is of great significance to increasing cotton yield and soil fertility. Therefore, this study aimed to isolate new phosphorus-solubilizing strains from saline soils in Xinjiang, China, to explore their effects on cotton growth, soil nutrients, and soil microorganisms, which can fill the gap of the interactions between saline soil-derived PSBs and cotton growth and yield.

We hypothesized that the PSBs isolated from salinized soils can increase cotton yield in lime-soil areas. In this study, PSBs that could solubilize phosphorus and produce indole acetic acid (IAA) were isolated from saline soils in Xinjiang, China, and identified. Then, different liquid bacterial suspensions were prepared and applied onto the cotton fields to clarify the influence of the selected PSBs on cotton growth, rhizosphere soil nutrients, and soil microorganisms. This study will deepen the understanding of the interaction mechanism between PSBs and plants in lime-soil systems and contribute to the sustainable agricultural development in arid areas.

## 2. Materials and Methods

### 2.1. Isolation and Identification of PSBs

#### 2.1.1. Culture Mediums

The NBRIP medium was prepared with 10 g of glucose, 5 g of Ca_3_(PO_4_)_2_, 5 g of sodium chloride, 1 g of (NH_4_)_2_SO_4_, 0.25 g of MnSO_4_·H_2_O, 5 g of MgSO_4_·7H_2_O, and deionized water (total volume: 1000 mL).

The LB medium was prepared with 10 g of peptone, 5 g of yeast extract paste, 10 g of sodium chloride, and deionized water (total volume: 1000 mL).

After adding 20 g of agar, the above culture mediums became solid culture mediums. In addition, we added 0.4% bromophenol blue solution to the NBRIP solid culture medium to assist observation.

#### 2.1.2. Isolation of PSBs

On March 20, 2023, plant samples were pulled out from salinized farmlands around Shihezi, Xinjiang, China, (44°18′ N, 86°3′ E) with an alcohol-sterilized shovel. After removing large pieces of soil attached to the root system, 500 g of rhizosphere soil attached to the root system were collected, put into sterile bags, sealed, stored at low temperature, and taken back to the lab.

The collected soil samples (10 g) were mixed with 3 g of NaCl in 90 mL of sterile water and shaken at 120 r·min^−1^ at 32 °C for 20 min, followed by the preparation of solutions with different dilutions (10^−1^, 10^−2^, 10^−3^, 10^−4^, and 10^−5^). Suspensions (0.1 mL) from 10^−3^, 10^−4^, and 10^−5^ tubes were cultured in the solid NBRIP medium at 32 °C for 3–5 days to observe and record the formation of a transparent circle. The formation of a clear transparent circle indicates the ability of the bacterial strains to solubilize inorganic phosphorus. A sterilized inoculating loop was used to pick up single colonies with an obvious transparent circle in the solid plate, and lines were drawn on the medium followed by observation. The strains were inoculated into the LB medium and stored in a refrigerator at 4 °C.

#### 2.1.3. Morphological and Molecular Identification of PSBs

The selected strains were cultured in the solid NBRIP medium to observe and record their characteristics, i.e., surface characteristics (smooth, rough, wrinkles, etc.), shape (concave, convex, and flat), size, color, transparency, edge characteristics (smooth, serrated, and wavy), etc.

Bacterial suspension (1 mL) was taken at the logarithmic growth phase (OD_600_ = 0.5–0.8) and centrifuged in a 1.5 mL sterilized centrifuge tube at 8000 r·min^−1^ at 4 °C for 2 min. After removing the supernatant, the bacterial cells were quickly frozen in liquid nitrogen for 15 min, stored at –80 °C, and then sent to Paisenuo Biotechnology Co., Ltd. (Shanghai, China), for identification. A phylogenetic tree was constructed using the MEGA 11 system based on the company’s identification results.

#### 2.1.4. Determination of the Phosphorus-Solubilizing Ability of PSBs

Each strain was inoculated into 20 mL of liquid LB medium and cultured on a shaker at 100 r·min^−1^ at 32 °C for 24 h. Then, the bacterial suspension was inoculated into the liquid NBRIP medium at 2% (V/V), cultured on a shaker at 100 r·min^−1^ at 32 °C for 5 d, and centrifuged at 8000 r·min^−1^ for 10 min. The soluble phosphorus concentration in the supernatant was measured using molybdenum antimony colorimetry [18].

The OD_700_ values of 0.0, 0.2, 0.4, 0.6, 0.8, and 1.0 mg/L of standard phosphorus solutions were measured, and the phosphorus standard curve was drawn based on the measured values. According to the standard curve equation and the absorbance value of the bacterial fermentation broths, the content of water-soluble phosphorus (PO_4_^3−^) in the PSB fermentation broths was calculated.

#### 2.1.5. Determination of Organic Acid Content and IAA Production

The suspensions of the bacterial strains were inoculated into liquid NBRIP medium at 1% (V/V) and cultured. The non-inoculated treatment was used as a blank control. Three replicates were set for each strain. After culturing on a shaker at 180 r·min^−1^ at 28 °C for 6 d, the culture medium was centrifuged at 8000 r·min^−1^ at 4 °C for 10 min, and the supernatant was collected and filtered through a 0.22 µm filter membrane. The types and contents of organic acids were determined using high-performance liquid chromatography (HPLC). A RIGOL L3000 (Saichang Scientific Instrument Co., Ltd., Jinan, China) high-performance liquid chromatograph and a Sapex HP C18 reverse-phase column (250 mm × 4.6 mm, 5 μm) were used. The mobile phase was prepared as follows: 1.56 g of sodium dihydrogen phosphate was dissolved in 800 mL of distilled water. After adding 16 mL of methanol, the pH of the solution was adjusted to 4–5 with phosphoric acid. The injection volume was 10 μL, the flow rate was 0.8 mL/min, the column temperature was 30 °C, and the detection wavelength was 214 nm.

Sackowski colorimetry was used to determine the ability of bacterial strains to secrete IAA [19]. The strains (n = 3) were separately inoculated into a liquid LB medium containing L-tryptophan and cultured in a constant-temperature shaking incubator at 180 r·min^−1^ at 30 °C for 3 d. The OD_600_ of the bacterial suspensions was adjusted to 1.0 with sterile water. The strain suspensions were then centrifuged at 4000 r·min^−1^ for 10 min. After transferring the supernatant (2 mL) into a tube, an equal amount of Sackowski color reagent was added. The mixture was shaken well and placed in the dark for 30 min. The OD_530_ value was measured spectrophotometrically to calculate the content of the IAA secreted by the strains.

#### 2.1.6. Determination of Enzyme Production by PSBs

The activities of acid phosphatase and phytase were measured to determine the phosphate-solubilizing and growth-promoting characteristics of the strains. The activity of acid phosphatase (μmol·min^−1^·mL^−1^) was determined using an acid phosphatase activity assay kit (Keming Biotechnology Co., Ltd., Suzhou, China) according to the instructions of the manufacturer. The activity of phytase (μmol·min^−1^·mL^−1^) was determined using a phytase activity assay kit (Keming Biotechnology Co., Ltd., Suzhou, China) according to the instructions of the manufacturer.

### 2.2. Experiment on PSBs’ Potential for Cotton Growth Promotion

#### 2.2.1. Experimental Site and Materials

From 20 April to 2 October 2023, the experiment was conducted in cotton fields at the Wulanwusu Ecological and Agricultural Meteorological Experimental Station in Xinjiang, China (44°17′ N, 85°49′ E, 468.2 m a.s.l.). This region has a temperate continental climate, with an annual average temperature of 7.2–8.0 °C, an annual average sunshine of 2700–2900 h, a frost-free period of 155–175 days, and an annual average precipitation of 235–275 mm (http://xj.cma.gov.cn/tzfm/shz/202205/t20220517_4834568.html, accessed on 30 April 2025). The soil type of the experimental site was gray desert soil, and the soil texture was sandy loam. The soil pH was 8.72, the organic matter content was 17.0 g/kg, the alkaline hydrolyzed nitrogen content was 15.4 mg/kg, the available phosphorus content was 16.8 mg/kg, and the available potassium content was 115.0 mg/kg.

The widely planted cotton variety in Xinjiang, Xinluzao 78, with a growth period of 115 days, was used in this experiment (provided by the Xinjiang Academy of Agricultural Reclamation Sciences, Shihezi, China).

The strains were separately inoculated into liquid LB medium, shaken for 24 h, and centrifuged at 8000 r·min^−1^ for 10 min. Then, sterile water was used to adjust the OD_600_ value to 1 to prepare the inoculant for application.

#### 2.2.2. Experimental Design

A total of four treatments were designed in this experiment, including (1) CK, which is conventional fertilization treatment (300 kg/hm^2^ of urea, 225 kg/hm^2^ of monoammonium phosphate, and 105 kg/hm^2^ of potassium sulfate); (2) P1, which is P1 inoculant treatment under conventional fertilization; (3) P2, which is P2 inoculant treatment under conventional fertilization; and (4) P3, which is P1 + P2 inoculant treatment under conventional fertilization. Each treatment had three replicates/plots. Each plot was 4 m wide and 8.5 m long. This experiment adopted a randomized complete block design, with the plots of each treatment not adjacent, i.e., the plots of different treatments were arranged in a staggered manner.

The experiment used drip irrigation and plastic film mulching in the planting of cotton, i.e., three drip tapes supplied water for six plant rows under one film. Cotton seeds were sown on 20 April 2023. The row spacing was 66 + 10 cm, and the plant spacing was 10 cm. Irrigation was conducted after sowing to promote seedling emergence. During the entire growth period, nine repeats of drip irrigation were conducted; 300 kg/hm^2^ of urea (N > 46%), 225 kg/hm^2^ of monoammonium phosphate (12−60−0), and 105 kg/hm^2^ of potassium sulfate (K_2_O ≥ 51%) were equally divided into nine parts and drip-fertigated through the drip irrigation system. Bacterial suspensions (OD_600_ = 1.0) were applied via the drip irrigation system (50 mL per fertilizer tank, 4.05 L for the entire growth period) after completing drip fertigation. Other managements were the same as those in local fields.

We collected soil (0−20 cm) and cotton plant samples during the flowering-bolling stage for the determination of soil physicochemical properties and soil metagenomics using multi-omics technology. Finally, we measured the cotton yield in October.

### 2.3. Test Methods

#### 2.3.1. Determination of Soil Properties

Soil pH, electrical conductivity, and the content of alkaline hydrolyzed nitrogen, available phosphorus, available potassium, and organic matter were determined as in [20].

#### 2.3.2. Soil Metagenomics

Rhizosphere soil samples (n = 3) were placed in an insulated box with ice packs and immediately brought back to the laboratory. After removing visible roots and stones, samples were sieved through a 2 mm sieve, stored at −80 °C, and sent to Personal Biotechnology Co., Ltd. (Shanghai, China) for sequencing based on the Illumina NovaSeq/HiSeq high-throughput sequencing platform.

#### 2.3.3. Determination of Cotton Growth Indicators and Yield

During the flowering-bolling stage, two cotton plants with similar growth status were selected from each plot (six plants per treatment). The fresh weight of each organ (cotton bolls, leaves, stems, and roots) and the root length were measured. After that, the samples were dried in an oven at 105 °C for 30 min and then dried at 90 °C to a constant weight, followed by weighing.

During the boll-opening stage, a quadrat was selected in each plot to measure the cotton plant density (6.67 m^2^ per quadrat). Then, six plants were selected in each quadrat, and the average number of bolls per plant was counted, followed by the measurement of the average weight of single bolls. Finally, the yield was calculated using the following formula, and the unit was converted to kg/hm^2^. The formula is as follows:

plant density × average number of bolls per plant × average single boll weight.

### 2.4. Data Analysis

Microsoft Excel 2021 was used to process data. The data were tested for normality in SPSS 27.0. The significance of differences was analyzed by analysis of variance (ANOVA), and Fishers’ least significant difference (LSD) was used for the multiple comparison of means (*p* < 0.05). Charts were drawn with Graphpad prism8 and Origin 2021. Correlation analysis and redundancy analysis (RDA) were conducted to investigate the relationship between soil microbial communities, cotton growth, and soil properties. A phylogenetic tree was constructed using the adjacency method in MEGA 11 based on the strain identification results.

## 3. Results

### 3.1. Morphological and Molecular Identification of PSBs

**Figure 1:**
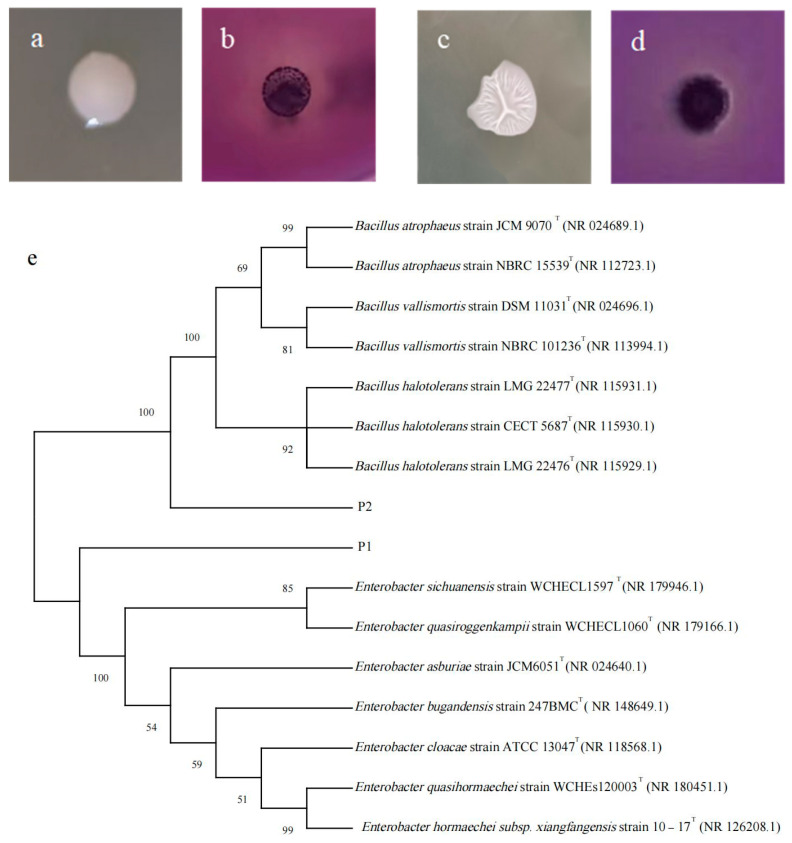
Morphology (**a**,**c**), phosphate-solubilizing circles (**b**,**d**), and phylogenetic tree (**e**) of phosphate-solubilizing bacteria P1 and P2. The symbol “T” indicates that the strain in question is the Type Strain.

Two strains with the colony morphology presenting as a transparent circle in NBRIP medium were extracted from the collected soil samples. The larger the circle, the higher the phosphorus-solubilizing efficiency. Figure 1b,d show the solubilizing effect of the two strains on Ca_3_(PO_4_)_2_. Phosphorus-solubilizing efficiency is related to the colony morphology. Figure 1a,c show the colony morphology of the strains inoculated into LB medium. The colonies of strain P1 were white, round, raised, and viscous, with neat edges. The colonies of strain P2 were white, with wrinkles on the surface and irregular edges.

According to the constructed phylogenetic tree (Figure 1e), strain P1 had a high similarity of 99.43% with *Enterobacter hormaechei*, and strain P2 had a high similarity of 99.93% with *Bacillus atrophaeus*. Thus, P1 was determined to be *E. hormaechei*, and P2 was *B. atrophaeus*.

### 3.2. Phosphate-Solubilizing and Growth-Promoting Ability of PSBs

**Figure 2:**
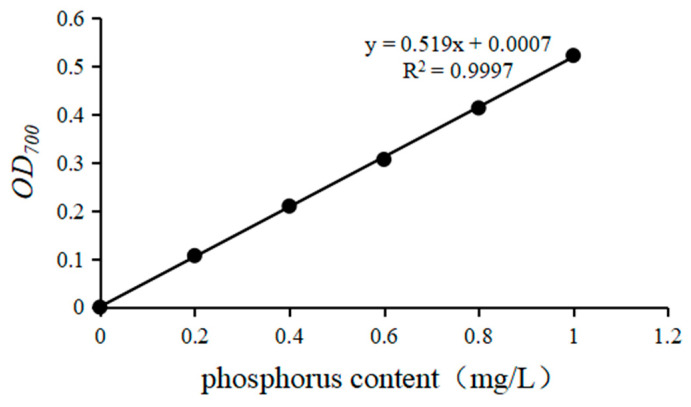
Standard curve of soluble phosphorus content.

The standard curve of soluble phosphorus content is shown in Figure 2. The linear regression equation was y = 0.519x + 0.0007, and the R^2^ was 0.9997. It can be seen that there is a good linear relationship.

The measurement results of the IAA concentration and enzyme activity (Table 1) showed that *E. Hormaechei* had a stronger ability to solubilize phosphorus than *B. Atropaeus*. Under the same culture conditions, the amount of phosphorus solubilized by *E. hormaechei* was 27.5% (635 mg/L) greater than that of *B. atropaeus* (*p* < 0.05). The IAA production in the *E. hormaechei* fermentation broth was 66.88% higher than that of the *B. Atropaeus* fermentation broth (*p* < 0.05). The activity of acid phosphatase and phytase in the *E. hormaechei* fermentation broth were 24.14% and 8.85% higher than those of the *B. Atropaeus* fermentation broth (*p* < 0.05).

**Figure 3:**
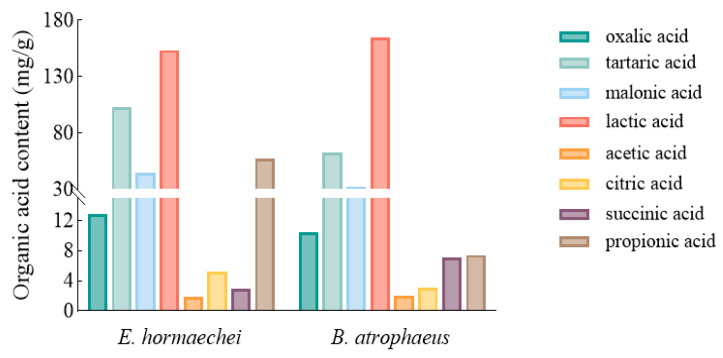
Types and concentrations of organic acids produced by phosphate-solubilizing bacteria *E. hormaechei* and *B. atrophaeus*.

Lactic acid (153 mg·g^−1^), tartaric acid (103 mg·g^−1^), and propionic acid (56.9 mg·g^−1^) were the main organic acids in the fermentation broth of *E. hormaechei.* However, lactic acid (164 mg·g^−1^), tartaric acid (62.6 mg·g^−1^), and malonic acid (32.5 mg·g^−1^) were the main organic acids in the fermentation broth of *B. atrophaeus* (Figure 3).

### 3.3. Effects of Phosphate-Solubilizing Bacteria on Bacterial Diversity and Community Structure in Cotton Rhizosphere Soil

**Figure 4:**
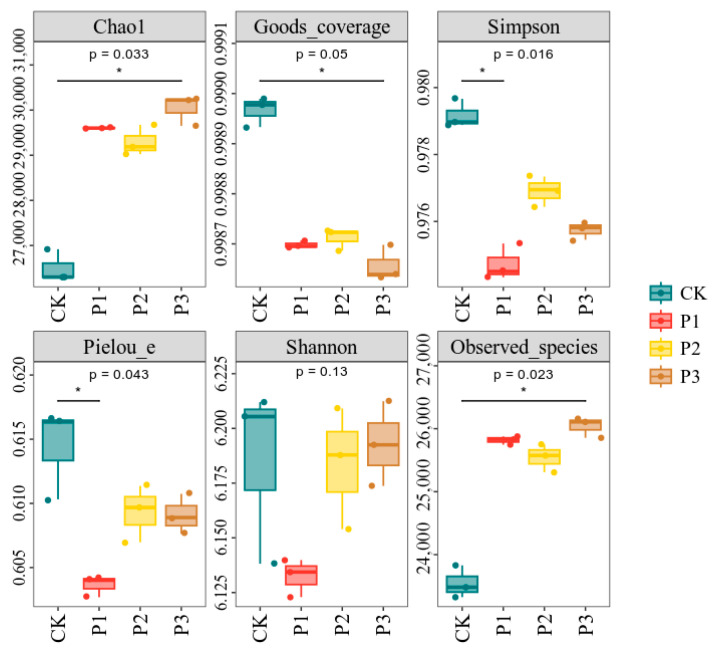
Soil microbial diversity indices under different treatments. *, *p* < 0.05.

The application of inoculants changed the Chao and Shannon indices of microorganisms in the rhizosphere soil of cotton. There were significant differences in the Chao1, Simpson, Pielou e, and observed species indices between the treatments (*p* < 0.05). In Addition, the differences in the Goods coverage and Shannon indexes were insignificant (*p* > 0.05). The soil microbial richness of the P1, P2, and P3 treatments significantly increased compared with that of CK, but the microbial coverage, diversity, and evenness significantly decreased (Figure 4). Among them, the decrease in microbial diversity and richness of the P1 treatment was most significant, and the microbial distribution was uneven.

**Figure 5:**
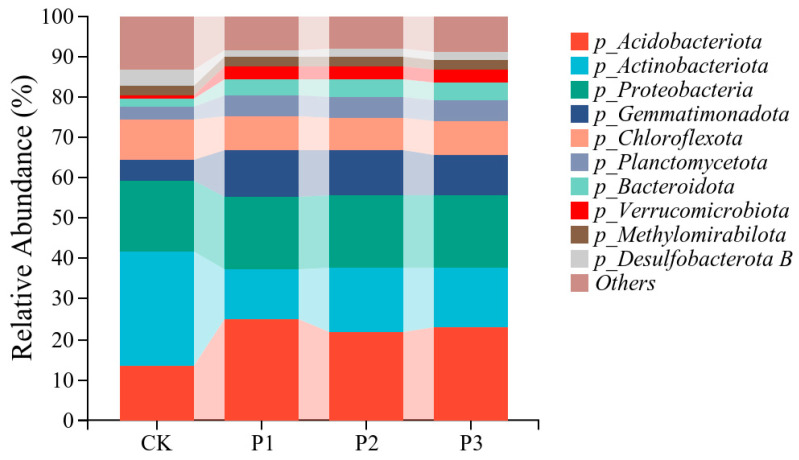
Effects of different treatments on microbial community structure in cotton rhizosphere soil at the phylum level.

*Acidobacteria*, *Actinobacteria*, *Proteobacteria*, *Gemmatimonadota*, *Chloroflexita*, *Planctomycota*, *Bacteroidota*, *Verrucomicrobota*, *Methylmirabilota*, and *Desulfobacteria B* were dominant bacterial phyla in the rhizosphere soils (Figure 5). These ten phyla accumulatively accounted for 86.93%, 91.8%, 91.9%, and 91.1% of the total bacterial community in the CK, P1, P2, and P3 treatments, respectively.

The application of PSBs changed the microbial community structure in rhizosphere soil (Figure 5). The abundances of *Acidobacteria*, *Gemmatimonadota*, *Planctomycota*, *Bacteroideta*, and *Verrucomicrobota* of the P1, P2, and P3 treatments significantly increased compared with those of the CK. Among them, the abundance of *Verrucomicrobota* increased the most, with an increase of 194%, 177%, and 170% in the P1, P2, and P3 treatments, respectively. The abundances of *Actinobacteria*, *Chloroflexita*, and *Desulfobacterata B* significantly decreased. Among them, *Desulfobacterata B* showed the largest decrease in abundance, with decreases of 57.8%, 47.8%, and 51.2% in the P1, P2, and P3 treatments, respectively.

### 3.4. Impact of Phosphate-Solubilizing Bacteria on Soil Properties

**Figure 6:**
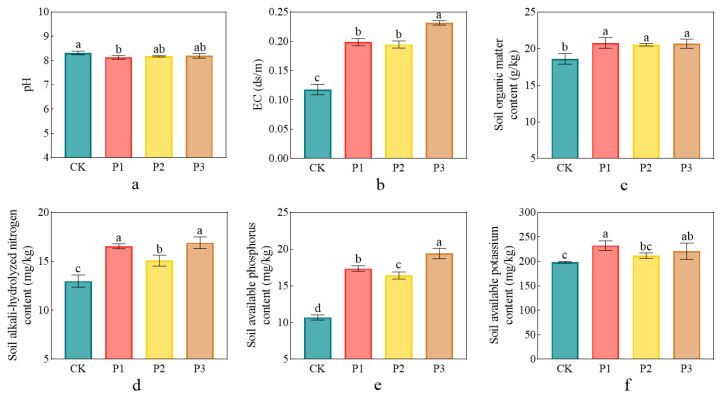
Effects of phosphorus-solubilizing bacteria on soil pH (**a**), conductivity (**b**), organic matter (**c**), soil alkali-hydrolyzed nitrogen (**d**), soil available phosphorus (**e**), and soil available potassium (**f**) contents. Different lowercase letters indicate significant difference between treatments at *p* < 0.05.

The application of PSBs improved soil physicochemical properties (Figure 6). Soil pH decreased after applying PSBs for a period of time, i.e., soil pH decreased from 8.31 (CK) to 8.13, 8.17, and 8.19 after applying P1, P2, and P3, respectively (Figure 6a). Soil conductivity of the P1, P2, and P3 treatments increased by 68.7%, 65.3%, and 96.7%, respectively, compared with that of CK (Figure 6b), and there was no significant difference between the P1 and P2 treatments.

The application of PSBs increased the available nutrient content in soil to varying degrees (Figure 6). The soil organic matter content of the P1, P2, and P3 treatments increased by 11.8%, 10.4%, and 11.2%, respectively, (*p* < 0.05) compared with that of CK (Figure 6c), but there was no significant difference between the three treatments. The soil alkaline hydrolyzed nitrogen content of the P1, P2, and P3 treatments increased by 19.7%, 16.1%, and 22.5%, respectively, (*p* < 0.05) (Figure 6d), the available phosphorus content increased by 57.4%, 45.7%, and 76.0%, respectively, (*p* < 0.05) (Figure 6e), and the available potassium content increased by 17.2%, 6.79%, and 11.3%, respectively, (*p* < 0.05) (Figure 6f), compared with those of CK.

### 3.5. Growth Promoting Effect of PSBs

**Figure 7:**
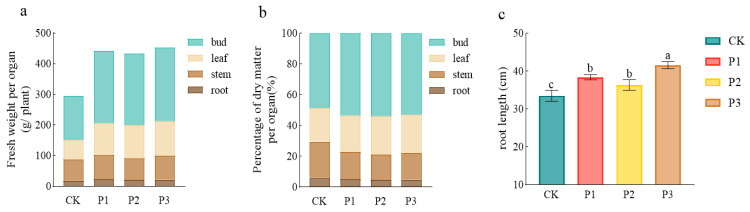
Effects of phosphate-solubilizing bacteria on fresh weight (**a**), dry matter accumulation (**b**), and root length (**c**) of cotton organs. Different lowercase letters indicate significant difference between treatments at *p* < 0.05.

The application of PSBs significantly promoted cotton growth (Figure 7). The total fresh weight of the P1, P2, and P3 treatments increased by 50.4%, 47.0%, and 54.1%, respectively, (*p* < 0.05) compared with that of CK, and there was no difference between the P1 and P3 treatments. Among all organs, the increase in boll weight was most significant. The boll weight of the P1, P2, and P3 treatments increased by 64.9%, 62.6%, and 67.7%, respectively, (*p* < 0.05), compared with that of CK (Figure 7a). The root length of the P1, P2, and P3 treatments significantly increased by 14.6%, 8.46%, and 24.1%, respectively, (*p* < 0.05), compared with that of CK, and there was no significant difference between the P1 and P2 treatments (Figure 7c).

**Figure 8:**
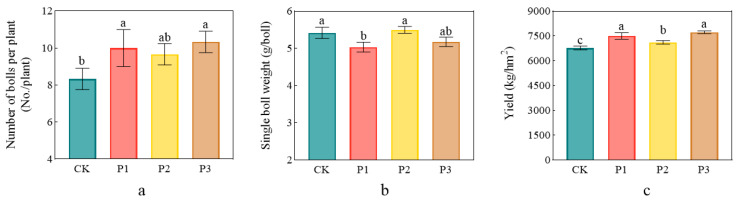
Effects of phosphorus-solubilizing bacteria on the number of bolls per plant (**a**), the weight of single boll (**b**), and cotton yield (**c**). Different lowercase letters indicate significant difference between the treatments at *p* < 0.05.

The application of PSBs significantly increased cotton yield compared with CK. The number of bolls per plant of the P1, P2, and P3 treatments increased by 20.0%, 16.0%, and 24.0%, respectively, (*p* < 0.05), compared with that of CK, and there was no difference between the P1 and P3 treatments. The boll weight of the P1 and P3 treatments decreased by 8.00% and 5.88%, respectively, (*p* < 0.05), compared with that of CK, and there was no difference between the P2 treatment and CK. The total yield of the P1, P2, and P3 treatments increased by 10.8%, 8.48%, and 14.0%, respectively, (*p* < 0.05), compared with that of CK, and there was no difference between the P1 and P3 treatments (Figure 8).

### 3.6. Correlation and Redundancy Analysis (RDA) Between Soil Microorganisms and Cotton Growth

**Figure 9:**
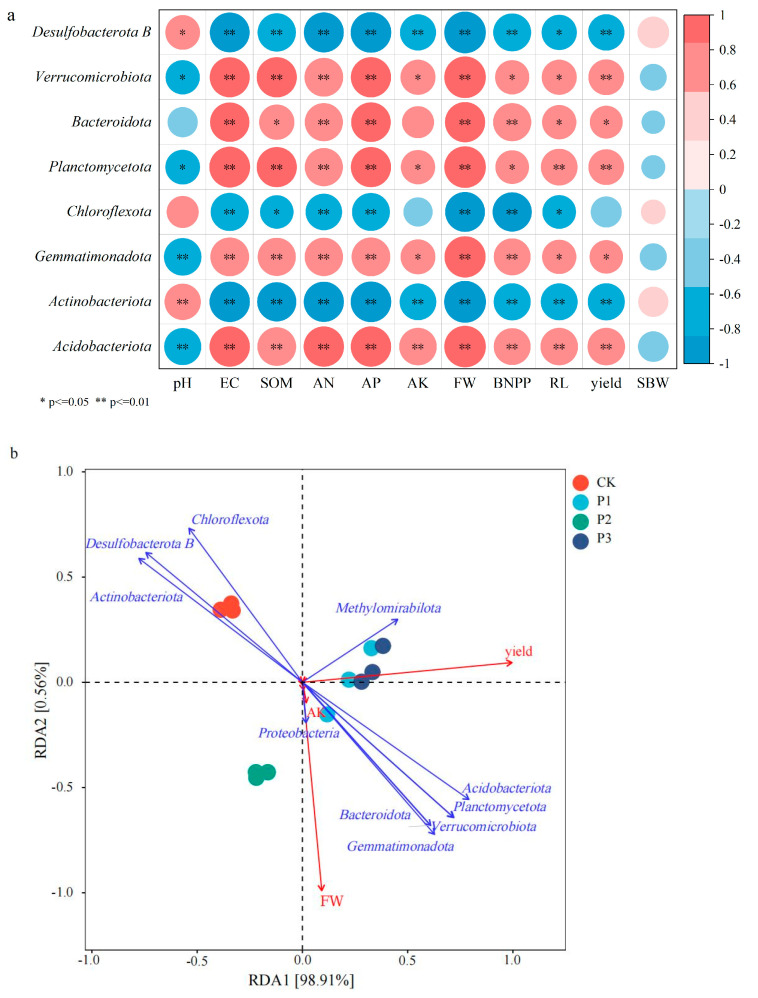
Correlation (**a**) and redundancy (**b**) analysis of soil microorganisms and cotton growth.

Except for *Actinobacteria*, *Chloroflexita*, and *Desulfobacterata B*, all other bacterial phyla were positively correlated with soil organic matter content, available nutrient content, cotton growth indicators, and yield (Figure 9a). Among them, *Acidobacteria* had the strongest correlation and *Desulfobacterata B* had the weakest correlation.

RDA 1 and RDA 2 explained 98.9% and 0.560% of the variation in the soil bacterial community structure, respectively. The soil bacterial community composition was significantly correlated with cotton yield and fresh weight, among which *Acidobacteria* and *Methylomyrabilota* were positively correlated with cotton yield, and *Proteobacteria* and *Bacteroidota* were positively correlated with cotton fresh weight and soil available potassium content (Figure 9b).

## 4. Discussion

*Enterobacter hormaechei* and *Bacillus atrophaeus* can transform insoluble phosphates into soluble phosphates by secreting organic acids and phosphatases, and they secrete IAA, promoting plant growth. Many existing reports only focus on their growth promoting effects and have not studied their organic acid production and enzymatic hydrolysis. For example, Bablesh Ranawat et al. [21] found that the salt-tolerant *Enterobacter hormaechei* could solubilize tricalcium phosphate into phosphate (99.7 μg/mL) and generate IAA (47.9 mg/L). However, in this study, the phosphorus solubilization capacity of P1 was 294 mg/L, which was higher than that of the strains isolated by Bablesh Ranawat et al. This may be due to the fact that the different organic acid production of the strains results in different phosphorus solubilization capacities. Sanchez Cruz et al. [22] isolated *Enterobacter hormaechei* subsp. Xiangfangensis from forest soil, and found that *E. hormaechei* had a maximum solubilizing capacity of tricalcium phosphate of 118 mg/L and a maximum secretion of IAA of 45.0 mg/L. Similarly, differences in phosphate-solubilizing capacity may also be due to differences in acid production and enzyme activity, or even differences in cultivation conditions. Xu et al. [23] found that the *B. atrophaeus* strain DX1708 extracted from the rhizosphere soil of chili could solubilize phosphate. Burak Alaylar et al. [24] identified one phosphate-solubilizing strain of *B. atrophaeus* in the Erzerum agricultural area. In this study, *B. atrophaeus* (P2) was extracted from saline soils in Xinjiang, China, showing good phosphate-solubilizing performance. *E. hormaechei* and *B. atrophaeus* extracted in this study could secrete organic acids, acid phosphatase, and phytase during phosphate solubilization. The organic acids and phosphatases produced by soil microorganisms are crucial for the cycling of inorganic and organic phosphorus in soil [25], and the phytase activity is closely related to microbial capacity to solubilize phosphorus [26]. It can be seen from Table 1 and Figure 3 that the acid-producing capability and related enzyme activities of *E. hormaechei* are higher than those of *B. atrophaeus*, and so the amount of phosphate solubilized by *E. hormaechei* is 27.48% higher than that of *B. atrophaeus*. These findings suggest the potential of *E. hormaechei* and *B. atrophaeus* in improving soil phosphorus availability and promoting crop growth.

The application of PSBs could significantly affect the microbial community structure and function in rhizosphere soil. Li et al. [27] showed that the inoculation with PSBs greatly impacted the structure and abundance of soil bacterial communities, although this did not significantly alter the bacterial α-diversity. In this study, the Chao index showed significant differences between the inoculant treatments (P1, P2, and P3) and CK, while there was no difference in Shannon index. This suggests that the application of PSBs may alter the abundance of some rhizosphere soil bacteria, but does not have a significant impact on the overall diversity. The differences in the change in Chao and Shannon indices after the application of PSBs may be due to the fact that the application of PSBs improves the supply of phosphorus in the soil, allowing some microorganisms that originally find it difficult to survive due to phosphorus limitations to grow and reproduce. However, these emerging microorganisms may have a small number in the community and are unevenly distributed, leading to the insignificant change in Shannon index. Wang et al. [28] found that inoculation with PSBs increased the abundances of *Proteobacteria*, *Chloroflexia*, and *Cyanobacteria* in the rhizosphere of Chinese cabbage compared with the CK. In this experiment, the application of PSBs increased the abundances of *Acidobacteria*, *Gemmatimonadota*, *Planctomycetota*, *Bacteroidota*, and *Verrucomicrobota* in cotton rhizosphere soil, but decreased the abundances of *Actinobacteria*, *Chloroflexita*, and *Desulfobactereota B*, compared with the CK. Studies have shown that *Acidobacteria* plays an important role in the carbon and nitrogen cycles, soil fertility increase, and cotton growth promotion [29]. *Acidobacteria* may have a certain antagonistic effect on the Verticillium wilt in cotton [30], so an increase in the abundance of *Acidobacteria* can enhance cotton’s resistance to pathogens. *Bacteroideta* can decompose complex organic matter, and both *Bacteroideta* and *Planctomycetota* can improve the carbon use efficiency of crops and play an important role in maintaining soil health [31,32]. Correlation analysis showed that *Actinobacteria*, *Chloroflexia*, and *Desulfobacteraceae* were negatively correlated with cotton growth indicators. The PSBs-induced decreases in the abundances of *Actinobacteria*, *Chloroflexia*, and *Desulfobacteraceae* reduce their potential negative impacts on cotton growth. Thus, it could be inferred that the application of PSBs can increase the number of microorganisms in the soil, while reducing their diversity and distribution range, allowing key microorganisms to play a better role.

The application of PSBs significantly impacted soil properties and cotton growth in this study. Rawat et al. [10] found that a decrease in the pH value of the culture medium promoted the solubilizing of insoluble inorganic phosphates. After the application of PSBs, the soil pH value decreased due to the organic acids produced during the metabolism of PSBs, which promoted the solubilizing of insoluble phosphates in the soil. In addition, it was found that the contents of alkaline hydrolyzed nitrogen, available phosphorus, and available potassium in the soil significantly increased after applying PSBs. This indicates that PSBs could promote nutrient cycling, phosphorus mineralization, and the release of potassium, increasing the availability of soil nutrients. Madhushita Pradhan et al. [33] found that inoculating with *B. amyloliquefaciens* CTC12 and *Burkholderia cepacia* KHD08 of the genus Bacillus significantly promoted the growth of peanuts and yield formation, bringing a yield increase of 114% and 113%, respectively, as well as a soil available phosphorus content increase of 65.0% and 58.0%, respectively, compared with the control. Mahdi et al. [34] found that the PSB *B. atrophaeus* GQJK17 S8 could increase germination rate, seedling biomass, and vitality index of *Chenopodium* quinoa plants compared with the control. Bablesh Ranawat et al. [21] found that *Enterobacter hormaechei* could fix N and solubilize a large amount of phosphorus and potassium, which promoted the change in the root structure and the growth of tomato plants, increasing tomato yield. In this study, the P3 treatment had the most significant phosphorus solubilizing and yield increasing effects compared with the control, followed by P1 and P2 treatments, indicating better performance of the mixed application of P1 and P2. Similarly, Iqra et al. [35] also found that the co-inoculation of IA6 and IA16 of the genus *Bacillus* increased the height, dry weight, root length, and leaf count of cotton plants compared with the control, showing better performance than the single application of IA6 or IA16. This yield-increasing effect is closely related to the improvement of the soil environment. *Enterobacter hormaechei* and *B. atrophaeus* could secrete organic acids to reduce soil pH, solubilize insoluble phosphates in the soil, and increase the bioavailability of soil nitrogen and potassium, promoting the growth and development of cotton roots and plants. The two PSBs work together to make the positive effects more significant. In addition, it was found that after the application of the inoculants the microbial community structure in the rhizosphere soil of cotton also changed. The abundances of *Acidobacteria*, *Bacteroidetes*, and *Fusarium* increased significantly, while those of *Actinobacteria* and *Chloroflexia* decreased. This indicates that the application of PSBs not only increases the available nutrient content in the soil, but also regulates the soil microbial community structure, further promoting the growth of cotton.

## 5. Conclusions

This study extracted *Enterobacter hormaechei* and *Bacillus atrophaeus* with strong phosphate-solubilizing ability from saline soil in Xinjiang, China. The two exhibited good phosphate-solubilizing performance and secreted organic acids and phosphatases. Field experiments showed that inoculation with the two phosphate-solubilizing bacteria had a significant impact on the microbial community structure in cotton rhizosphere soil, i.e., increasing the abundance of beneficial bacteria and reducing the abundance of unbeneficial bacteria. In addition, it also significantly reduced soil pH and increased the contents of soil alkaline hydrolyzed nitrogen, available phosphorus, and available potassium, enhancing soil fertility. These jointly promote cotton growth and yield formation. The research results show that phosphate-solubilizing bacteria do exist in salinized soils and could increase cotton yield in low-phosphorus lime-soil areas. This study provides new strategies for sustainable agricultural development. However, there are also some limitations in this experiment. This is a one-year study conducted in a single location. In the future, the effects of PSBs can be further explored for multiple years and on different crops in different soil types. In addition, the application potential of PSBs in organic fertilizers and ecological agriculture can be studied in depth to provide technical support for the development of green agriculture.

## Figures and Tables

**Table 1:** Phosphate-solubilizing and growth-promoting ability of *Enterobacter hormaechei* and *Bacillus atrophaeus*.

Strain	Soluble Phosphorus Content (mg·L^−1^)	Indoleacetic Acid Content (ng·mL^−1^)	Acid Phosphatase Activity (μmol·min^−1^·mL^−1^)	Phytase Activity (μmol·min^−1^·mL^−1^)
*E*. *hormaechei*	294 ± 8.50 a	359 ± 0.89 a	0.293 ± 0.002 a	0.289 ± 0.0012 a
*B*. *atrophaeus*	231 ± 4.25 b	215 ± 0.59 b	0.230 ± 0.001 b	0.262 ± 0.0004 b

Note: different lowercase letters indicate significant difference between treatments at *p* < 0.05.

## Data Availability

The data presented in this study are available at reasonable request to the corresponding author.
